# The X Factor in Immunity: Sex Differences Shaped by the X Chromosome

**DOI:** 10.1111/imr.70107

**Published:** 2026-01-23

**Authors:** Katherine B. Radovanovic, Megan Wynalda, Montserrat C. Anguera

**Affiliations:** ^1^ Department of Biomedical Sciences, School of Veterinary Medicine University of Pennsylvania Philadelphia Pennsylvania USA

**Keywords:** autoimmunity, immune signaling, sex differences, viral infections, X‐chromosome inactivation, Xist RNA

## Abstract

There are sex differences with immune responses where females exhibit stronger immune responses compared to males. Both sex hormones and sex chromosome differences between males and females contribute to the observed sex differences with innate and adaptive immune cell composition and function. Here, we present recent findings investigating sex differences with immune cells and responses, highlighting contributions from sex hormones or X‐linked genes. We also review the epigenetic gene regulatory mechanisms that form the inactive X chromosome (Xi), known as X‐Chromosome Inactivation (XCI), the heterochromatic nature of the Xi that allows for selective gene reactivation, and recent work investigating the mechanisms of XCI maintenance in female immune cells. We also highlight some dosage‐sensitive X‐linked genes involved in female‐biased autoimmune disease, discuss sex differences with immune signaling pathways, and sex differences with viral infections and vaccine responses with the ultimate goal to leverage these insights for the development of sex‐specific therapeutic interventions for immune‐related diseases.

## 1 Introduction

Immune responses are influenced by biological sex differences between males and females, and these differences originate from the sex hormones and sex chromosomes. Human males and females differ not only in the overall abundance of circulating immune cells but also in the proportional distribution across immune subsets. Sex differences reflect a broader narrative of immune dimorphism: females generally mount stronger adaptive responses, while males tend towards heightened innate and inflammatory activity. Studies of influenza vaccination responses show that females exhibit stronger adaptive [1], humoral [2], and cellular [3] responses. Sex hormones, specifically estrogen and androgens, vary in concentration over the lifespan and can have pro‐inflammatory or anti‐inflammatory effects, as hormones bound to nuclear hormone receptors regulate the expression of many immune genes. The X chromosome is a genetic and epigenetic contributor to sex‐specific gene expression in immune cells and contains many important immune‐related genes, some with known dosage sensitivity. Both sex hormones and sex chromosomes contribute to observed sex differences with immune‐related diseases, especially autoimmune diseases that typically affect more female individuals.

Females exhibit greater humoral responses, characterized by higher numbers of B cells and increased levels of antibodies, which may contribute to their stronger vaccine responses and increased susceptibility to autoimmune disorders [4]. Yet, males tend to have greater monocyte and neutrophil activity, supporting stronger innate inflammatory responses but leaving them more susceptible to severe outcomes following pathogen infection. Despite these patterns, gaps remain in our understanding of the mechanisms underlying these sex‐based differences in immune responses. Most studies focus on immune cell subsets in circulation, while less is known about sex differences in tissue‐resident immune cells, how quantities of immune cells differ within tissues, and how immune responses may differ in specific tissues depending on biological sex. Moreover, the role of genetics and epigenetic regulation in shaping the immune landscapes of autosomal and sex‐linked gene expression is not well understood. Addressing these gaps will be crucial for translating observed cellular differences into mechanistic insight, and ultimately, for tailoring sex‐specific approaches to immunotherapy, vaccination, and chronic disease management.

## 2 Sex Differences in Immune Cell Composition and Function

### 2.1 Sex Differences With Human Immune Cells in Circulation

Peripheral blood analyses allow for robust comparisons of immune cell subsets across large populations and sampling over time has revealed consistent observations of sex differences with immune composition. Examining peripheral immune cell compositions has also yielded insight into how these sex differences in immune cells might contribute to sex‐specific risks for immune diseases and outcomes. Multiple studies across independent cohorts have observed that human females have higher numbers of CD4+ and CD8+ T cells, as well as proliferating T cells following stimulation of PBMCs [5, 6]. Females also have proportionally higher numbers of naïve CD4+ T cells [7, 8, 9] and lower frequencies of natural killer (NK) cells [5, 7, 8, 9, 10] compared to males. Human females also have higher frequencies of CD25+ CD127− regulatory T cells [8] and mucosa‐associated invariant T cells in circulation [9]. Additionally, females have been noted to have higher frequencies of CD19+ B cells [5, 8], plasma cells [10], and higher IgM concentrations [11], in support of a female bias with humoral immunity. On the other hand, human males have higher levels of CD14+ and CD16+ monocytes [12], suggestive of a more pro‐inflammatory state. Moreover, males often exhibit higher neutrophil‐to‐lymphocyte ratios [13], which is used as a clinical marker of systemic inflammatory balance [14], further supporting a more inflammation‐prone baseline immunological state.

### 2.2 Sex Differences With Human Tissue‐Resident Immune Cells

Sex differences are also present in the composition and behavior of immune cells within tissues, which are critical for homeostasis and mounting localized immune responses to infection or injuries [15]. There are sex differences in the quantity of immune cells, which adds to the complexity of understanding systemic immune regulatory pathways as tissue resident immune cells influence inflammation pathways. Thus, examining sex‐specific immune cell composition within tissues is essential for understanding sex‐biased disease manifestations in organ‐specific contexts as well as differences in how the immune system responds to pathogens or inflammatory disease states.

One of the clearest examples of sex‐based differences in tissue‐resident immune cells is in lymphoid tissues, which serve as key hubs for antigen presentation and adaptive immune cell activation. In tonsillar tissue, females display higher numbers of memory B cells compared to males, a difference that persists across age groups [16]. Furthermore, B cell subsets marked by CD21, CD45RB, CD73, and CD39 are significantly enriched in female compared to male tonsils. These findings suggest that females maintain a more robust pool of memory and regulatory B cells within secondary lymphoid organs. On the one hand, this could enhance immune recall responses and provide stronger protection against infection (and repeat infections), but it may predispose females to greater immune activation, which is a feature of autoimmune disease. While tonsillar data represent one of the few detailed examinations of sex‐specific tissue immunity, studies examining sex differences in other lymphoid tissues such as lymph nodes and spleen remain incredibly scarce, and comparable data in non‐lymphoid organs are limited as well. This gap highlights a need to map tissue‐specific immune landscapes across sexes, which would help provide a more complete understanding of how local immune environments contribute to sex‐biased health and disease.

There are inherent challenges to studying the immunological foundation of human tissues, including limited access to samples, heterogeneity between tissue microenvironments, and technical constraints in capturing dynamic immune interactions. With recent advances in sampling and profiling techniques, such as single‐cell transcriptomics, spectral flow cytometry, and spatial imaging, it is becoming possible to achieve more detailed characterizations of these sex‐specific patterns [15]. These studies will have profound implications on future development of sex‐specific immunological interventions and therapies.

### 2.3 Sex Differences With Immune Cell Composition in Mouse Models

PBMCs from female C57BL/6J mice exhibit higher proportions of CD4+ and CD8+ T cells and CD19+ B cells, and lower proportions of CD11b+ Ly6B‐Ly6C+ monocytes, CD11cb+ Ly6G+ Ly6c+ neutrophils, and CD11b^mid^ NK1.1+ NK cells compared to males [17]. These sex‐specific trends mirror those observed in human blood, yet there are sex differences in immune cell composition when comparing human and mouse studies. Although male mice display greater NK cell numbers in the spleen, their cytotoxic function is impaired because of reduced expression of the X‐linked histone lysine demethylase gene *Kdm6a* [18]. This finding illustrates how cell‐intrinsic regulation, particularly involving X‐linked genes, can shape functional outcomes irrespective of absolute cell number.

Mice also exhibit sex differences in tissue‐resident immune cells. Female mice have greater numbers of F4/80+ macrophages, CD4+ and CD8+ T cells, and CD19+ B cells in peritoneal and pleural cavities [19]. The proportion of CD3+ cells in the pleural and peritoneal cavities is greater in females, yet the frequencies of F4/80+ macrophages and CD19+ B cells are similar, perhaps due to sex differences with chemotaxis [19]. Work using mice and human samples has revealed that activation of NK cells depends on CD4+ T cell‐mediated secretion of IL‐12 [20], and that sex‐specific differences with CD8+ T cell responses can also influence IL‐12 production [21]. Thus, IL‐12 can mediate T cell‐NK cell communication and also contributes to the observed sex differences with T cells. Additionally, innate lymphoid cells (ILCs), which coordinate immune responses through effector cytokines, are reduced in the lungs of female mice compared to males [4]. This skew has been implicated in female‐biased susceptibility for multiple sclerosis [22] and asthma [23, 24]. Male mice where androgen has been depleted using orchiectomy experiments exhibit reduced numbers of ILC2 cells, suggesting that testosterone influences increased ILC2 numbers [17]. These observations underscore the importance of studying immune compartments beyond circulation, as localized environments may amplify or attenuate sex biases.

A concern when using female mice is that hormonal fluctuations, particularly across the estrous cycle, may be a confounding factor. However, one study demonstrated that variability in female mice is no greater than in males [25], reinforcing the reliability of female animals in immunological research. This has important implications, as it challenges long‐standing biases that historically favored male mice in preclinical studies. Together, these findings underscore the dual value and complexity of murine models as they provide mechanistic insights into sex‐specific immune regulation that cannot be easily obtained in humans yet also reveal layers of gene–environment–sex interactions that should be carefully considered.

## 3 Origins for Sex Differences: Sex Hormones

Sex differences arise in part from differing endocrine environments that are established during development and maintained throughout life. Two prominent members of the steroid hormone family, estrogen and testosterone, serve as central regulators of the differentiation, maturation, and function of many reproductive and non‐reproductive tissues and can also influence the immune system. Although genetic factors, including X‐linked immune regulators and X‐linked genes expressed from the inactive X (Xi), contribute to sex differences in immune responses (discussed in the following section), the sex bias of many autoimmune diseases is influenced by the immunomodulatory effects that sex hormones exert on innate and adaptive immune pathways across the lifespan.

Estrogen acts through estrogen receptors alpha and beta (ERα and ERβ), which are expressed on a wide variety of immune cells, including dendritic cells, B cells, T cells, and macrophages. ERα and ERβ bind to hormone response elements within promoter regions to regulate gene expression. Estrogen can shift macrophage polarization to repair damaged tissues, and the macrophage M2‐like phenotype is characterized by increased ornithine production, which supports cell proliferation, tissue repair, and anti‐inflammatory properties [26, 27, 28, 29, 30]. Estrogen also modulates helper T cell fate where elevated estrogen levels, such as those observed during pregnancy, can promote Th2 responses that favor immune‐mediated repair [31, 32] and antibody‐mediated responses over the more pro‐inflammatory Th1 and Th17 pathways. Sex hormonal transitions across the lifespan, including puberty, pregnancy, menopause, and exogenous hormone treatment, are associated with distinct immune phenotypes and disease susceptibilities [33, 34]. Elevated estrogen contributes to heightened responsiveness to vaccination and pathogen infection and also exhibits protective effects such as improved survival in sepsis [35]. This context‐dependent nature of estrogen highlights its complex role as both a potential protector and promoter of autoimmunity, depending on concentration, timing, receptor distribution, and the immune microenvironment. In both human and mouse models of systemic lupus erythematosus (SLE), B cells are highly sensitive to estrogen levels [36, 37] and increased estrogen results in elevated production of IgG, IgM, and anti‐dsDNA autoantibodies [38].

In contrast to estrogen's predominantly stimulatory and reparative influence on the immune system, the androgens testosterone and dihydrotestosterone often exert immunosuppressive effects [39, 40, 41, 42]. Androgen receptor signaling generally dampens pro‐inflammatory responses in many immune compartments [43]. In innate immune cells, androgens can alter signal transduction through pattern recognition receptors to downregulate NF‐kB and Toll‐like Receptor (TLR4) expression, and orchiectomized mice treated with exogenous testosterone can reverse the elevated TLR4 expression level [44]. Androgens also reduce pro‐inflammatory cytokine production by monocytes and macrophages. Androgens can inhibit immune and humoral responses by reducing lymphocyte proliferation, cytokine and immunoglobulin production, and blocking development of B cell progenitors [43]. In the spontaneous mouse model of lupus‐like disease, NZB/W F1 mice, testosterone regulates the expansion of immunosuppressive neutrophil subsets that confers protection against lupus‐like disease manifestations [45]. Androgens can also suppress Th1 differentiation, providing a mechanistic explanation for the lower male prevalence of Th‐1 mediated autoimmune pathologies such as type 1 diabetes [43].

Taken together, estrogen and testosterone exert complementary yet contrasting effects on the immune system. While estrogen enhances antibody production, promotes B‐cell survival, and shifts immune responses towards humoral pathways, sometimes at the cost of increased autoimmune susceptibility, testosterone generally reduces inflammatory signaling, limits effector T‐cell expansion, and strengthens immunoregulatory networks. The interplay of these hormonal effects contributes to the pronounced sex differences in autoimmune disease incidence and clinical presentation, underscoring the importance of incorporating endocrinological foundation in models of immune dysregulation and pathology.

## 4 Origins for Sex Differences: Formation of the Inactive X Chromosome (Xi) and Gene Regulation From the Xi

The X chromosome contains many genes critical to immune function [46], and increased expression of some X‐linked immunity‐related genes is a feature of various female‐biased autoimmune diseases [47]. Female mammals with two X chromosomes utilize a dosage compensation mechanism that enables appropriate expression levels of X‐linked genes. This mechanism is X‐Chromosome Inactivation (XCI), where one X chromosome is randomly selected early in embryonic development to undergo transcriptional silencing across most, but not all, of the chromosome. The Xi is mostly transcriptionally silent in female immune cells, yet there are developmental stages where female cells can tolerate a fully‐reactivated Xi. In this section, we review how the inactive X (Xi) is formed during early embryonic development and examine recent work investigating mechanisms of gene regulation across the Xi in female T and B cells. Ultimately, exploring the epigenetic and silencing mechanisms during the formation of the Xi provides insight into the pathways that regulate Xi‐linked gene expression in immune cells and further strengthens our understanding of the context surrounding immune cell function.

### 4.1 The Inactive X Chromosome Is Facultative Heterochromatin, Capable of Gene Reactivation

The Xi is a classic example of facultative heterochromatin, which can be reactivated during specific developmental contexts. After fertilization in mice, the female zygote inherits a somewhat transcriptionally silent paternal X chromosome (Xp). The origins of Xp silencing occur in the male germline, which is observed across eutherian mammals and also worms [48]. During the first meiotic prophase of spermatogenesis, the X chromosome and Y chromosome pair together and are transcriptionally silenced in a process called Meiotic Sex Chromosome Inactivation [48, 49]. The X chromosome acquires heterochromatic histone tail modifications [48, 49], and most X‐linked genes become transcriptionally silent yet about 15% are expressed from the somewhat silent paternal X chromosome (Xp) [49], as the X chromosome contains many genes important for spermiogenesis [48]. While the zygote undergoes cell division, the maternal X chromosome (Xm) remains active and the Xp becomes more silent, occurring first at repetitive regions followed by gene regions [50], and by the morula stage the Xp is mostly silent. At the blastocyst stage, the extraembryonic cells, which will form the placenta, exhibit imprinted XCI, where the Xp is silent [48]. The primitive endoderm of the female blastocyst, which becomes the yolk sack, also exhibits imprinted XCI. However, the Xp of the epiblast becomes fully reactivated in preimplantation female blastocysts, and these pluripotent cells contain two transcriptionally active X chromosomes. The pluripotent state is remarkable because it can tolerate the double transcriptional dosage of X‐linked genes, unlike most somatic cells examined to date [51, 52]. Xp reactivation is initially restricted to epiblast cells expressing the pluripotency transcription factor NANOG, where *Xist* is transcriptionally downregulated, along with reduced Polycomb Repressive Complexes (PRCs) activity and less H3K27me3 and H3K9me3 enrichment on the Xp. As the female embryo implants and undergoes gastrulation, each epiblast cell will randomly select one X for transcriptional silencing, known as random XCI, to convert the active X (Xa) into an epigenetically distinct and mostly transcriptionally silent Xi. The extraembryonic mesoderm compartment of the yolk sac, which is the origin of the hematopoiesis and the immune system, also exhibits random XCI [53]. Random selection of chromosomal silencing gives females an evolutionary advantage, with cells expressing genes from both the Xp and Xm, creating mosaicism in female X‐linked gene expression. Female mosaicism, especially in the context of immune function, protects females from X‐linked gene mutations, increases the diversity of available cellular proteins, enhances viral immune responses, and contributes to the overall survival outcomes across the female lifespan.

Random XCI begins with upregulation of the long noncoding RNA called Xist from the future Xi. Xist RNA is one of the most studied long noncoding RNAs [54] and is necessary for both Imprinted and Random XCI. *Xist* upregulation on one X chromosome triggers a cascade of epigenetic activity on the future Xi [55]. The active histone modification H3K27acetyl [56] and occupancy of RNA Polymerase II [57] are reduced from the future Xi. Heterochromatic histone tail modifications H3K27me3 [56, 58, 59, 60], H2AK119‐Ub [56, 61], and H3K9me3 [62, 63] become enriched chromosome‐wide by Polycomb Repressive Complexes (PRC) 1, 2, and SETDB1. The Xi also becomes enriched with the histone variant macroH2A and DNA methylation (DNAm) at both CpG islands and promoter regions [64, 65, 66].

### 4.2 Mechanisms That “Count” and “Choose” an X Chromosome for Repression in Female Embryonic Stem Cells

XCI initiation occurs in genetically distinct steps where the cell can “count” the number of X's present in a diploid nucleus, then “choose” to silence all X's except one, orchestrated through precise management of X‐linked and autosomal regulatory elements [67]. The “counting” phenomenon is the calculation of the X‐to‐autosome ratio (X:A) and follows the “*n* − 1” rule where all but one X is silenced per diploid nucleus [68, 69, 70, 71]. Thus, XO and XY cells with X:A = 0.5 will not silence the X, whereas XX and XXY cells have X:A > 1.0 and will silence 1 X. Using X chromosome structural variants, the master control center for XCI initiation was discovered within a 1‐Mb region of human Xq13‐21 [72, 73], named the X Inactivation Center (*XIC*), which regulates every step of XCI: counting, random choice, and initiation of transcriptional silencing. The mechanism of counting is still unresolved [67], yet likely involves two factors: a “blocking factor” which binds to and blocks the *XIC* for protection of one X from inactivation [68, 69, 74] and a “competency factor”, likely produced at the *XIC* by the 2nd X in female cells (and missing in XY cells), which functions for XCI initiation [75, 76]. It has been proposed that the CTCF protein is the autosomal blocking factor and that the long noncoding RNA Jpx, located within the *XIC*, is the X‐linked competency factor. CTCF protein and Jpx RNA can bind together, possibly in a functionally antagonistic fashion, to regulate the process of counting X chromosomes, although more work is needed to test this mechanism [77].

The “choice” step of XCI, to inactivate either Xm or Xp, is random, and the mechanism occurs quickly and in a mutually exclusive and irreversible fashion. “Choice” requires two noncoding RNAs at the *XIC* region: the antisense RNA Tsix, which overlaps the *Xist* gene, and Xite RNA, which is an enhancer of *Tsix* [78]. In female epiblast cells with two Xa, before XCI begins, Tsix RNA is abundantly expressed from both X chromosomes, preventing *Xist* upregulation [76, 78, 79, 80]. At the onset of XCI initiation, the two Xa transiently pair together at the *XIC*, and after they come apart, one X continues to express *Tsix* and will become the future Xa and the other expresses *Xist*, ready to become the Xi [81, 82, 83]. The pairing of the two X chromosomes enables crosstalk between the two identical alleles that primes opposite and mutually‐exclusive fates for each X. On the future Xa, expression of the *Xite* enhancer allows persistent *Tsix* expression which prevents Xist RNA upregulation; thus Tsix RNA is essential for allelic choice. After pairing, the future Xi is specifically enriched with CTCF proteins across the *Tsix* and *Xite* regions [82, 83], which act as transcriptional repressors for *Tsix* and *Xite* thus allowing for Xist upregulation on the future Xi. Additional work is needed to determine how the two Xs communicate when they are paired together, and what regulates the asymmetric distribution of CTCF protein binding across the *Tsix* and *Xite* regions.

### 4.3 Xist RNA: The Long Noncoding RNA That Regulates Gene Expression on the Inactive X Chromosome

Xist RNA is a crucial component of both random and imprinted XCI, and it is also important for XCI maintenance in female somatic cells including those of the immune system [84, 85]. *Xist* is mono‐allelically expressed from the Xi and becomes upregulated from the future Xi after the choice step is completed [86]. Xist RNA transcripts range in size from 17 to 20 kB, and they are spliced and polyadenylated. Xist RNA is retained in the nucleus and usually localizes to the Xi in *cis* in most female cells [87]. Inducible XIST transgenes, either the *XIST* gene, XIST cDNA, or the *XIC* region, are sufficient to initiate transcriptional repression of the chromosome from which it is expressed, even if it is located on an autosome [51, 88, 89]. Like most long noncoding RNAs, Xist RNA contains various repeat domains that are evolutionarily conserved across eutherian mammals, known as Repeats A‐F [86]. Each of the Xist RNA repeat regions can recruit specific proteins or enzymatic factors to perform various functions: recruitment of repressive chromatin complexes, eviction of activating complexes, and changes with X chromosome ultrastructure. Xist RNA Repeat F functions as the nucleation site for Xist RNA to bind to the Xi, prior to Xist RNA spreading, where the transcription factor Yin Yang 1 (YY1) can bind to tether Xist RNA to the Xi [67, 90]. YY1 acts across the genome in early development, cellular differentiation and proliferation through its ability to activate or repress transcriptional activity [91], yet also tethers Xist RNA to the future Xi at Repeat F sites during XCI initiation. The Xist RNA Repeats A and B are associated with gene silencing and PRC2 recruitment [92]. The Xist Repeat A region is necessary for transcriptional silencing [93, 94], yet deletion of Repeat B also results in impaired, yet less severe, Xi silencing [95]. Xist Repeat A recruits PRC2, where Xist RNA binds to the EZH2 catalytic subunit of PRC2 [67, 96, 97], although the exact structural conformation of the Repeat A is still under investigation [94, 98]. Repeat B can also recruit PRC2 [92, 96, 99]. Repeats B and E of Xist RNA are required for spreading Xist RNA transcripts across the Xi during XCI initiation [86, 95, 100, 101]. Deletion of either Repeat B or Repeat E results in dispersed Xist RNA “clouds” at the cytological level as visualized by RNA Fluorescence in situ Hybridization.

Using RNA mapping techniques for molecular resolution of Xist RNA occupancy across the Xi during XCI initiation, Xist RNA transcripts first accumulate at the *Xist* gene then spread to active X‐linked genes in three dimensions across the Xi, eventually covering the entire X [60, 102]. Repeat B of Xist RNA is necessary for Xist RNA spreading and silencing of some X‐linked genes [95]. PRC2 and YY1 are co‐transcriptionally recruited by Xist RNA to the Xist gene region of Xi during nucleation, while Xa binding sites are blocked [67]. The current model for the origins of Xist RNA spreading starts with Xist RNA, YY1, and the PRC2 complex recruitment to the nucleation center, which triggers the spread of Xist RNA in *cis* along the Xi [67]. Xist RNA also induces spatial reorganization of the newly formed Xi into 2 “mega‐domains”, and Repeat B is necessary for attenuating topologically associated domains (TADs) across the Xi during XCI initiation. Thus, Xist RNA can change the 3D ultrastructure of the Xi through interactions with the SMCHD1 protein, which merges compartments within each mega‐domain, and SMCHD1 also facilitates the regional spreading of both Xist RNA and H3K27me3 accumulation across the Xi [103, 104].

### 4.4 Gene Expression From the Inactive X Chromosome: XCI “Escape” Genes

It is important to note that although the Xi is mostly transcriptionally silent, there are genes that are expressed from the Xi, known as XCI “escape” genes [105]. *Xist* is one XCI escape gene, and *Xist* is consistently and uniquely expressed from the Xi in all female immune cells examined to date. XCI escape genes exhibit variability across tissues and organs, and also between individuals [105]. About 15% of expressed X‐linked genes in humans exhibit XCI escape, and the numbers are lower in mouse somatic cells [106]. Often, XCI escape genes account for sex differences with gene expression between XY males and XX females, who would otherwise only have transcriptional activity from one chromosome [107]. The pseudoautosomal regions (PAR1 and PAR2), which are identical between the X and Y chromosomes, are two regions with high levels of XCI escape [107]. All genes contained within the human Xp PAR1, along with genes on the distal ends of the Xq PAR2 regions, are known to escape XCI [108, 109, 110, 111]. XY homology gene pairs are also constitutive XCI escape genes [107], and these genes are dosage‐sensitive and highly evolutionarily conserved [112]. Expression of XCI escape genes is much lower from the Xi compared to the Xa, which underscores their dosage sensitivity and also the presence of allele‐specific repressive mechanisms that prevent equivalent expression as from the Xa [113, 114]. Although XCI escape genes exhibit low expression levels from the Xi, their expression is required for development and normal cellular function. Patients with Turner syndrome are mosaic for cells that contain one X chromosome (monosomy X; 45, X) [115], and full monosomy X is embryonic lethal in 99% of female embryos because of impaired placental differentiation [116, 117]. Turner syndrome patients who survive gestation exhibit short stature, reduced or absent fertility, ovarian dysgenesis, impaired intellectual development [118], with severity depending on the degree of mosaicism and the loss of XCI escape gene expression [116]. Epidemiological studies have revealed that Turner patients also have an increased risk of developing autoimmune thyroid disease, in particular Hashimoto's thyroiditis, celiac disease, type 1 diabetes, and inflammatory bowel disease [119]. The increased susceptibility of Turner syndrome patients to these autoimmune diseases may be caused by haploinsufficiency of some XCI escape genes, but these associations have not been carefully investigated.

### 4.5 Female T and B Cells Use Dynamic Localization of Xist RNA and Heterochromatic Marks to Regulate X‐Linked Gene Expression From the Xi

Female lymphocytes, unlike most female somatic cells, exhibit differences with XCI maintenance mechanisms, where canonical Xist RNA “clouds” and foci for heterochromatic histone modifications are absent from the Xi in naïve lymphocytes [120, 121, 122]. Following antigen stimulation, Xist RNA clouds and heterochromatic histone marks become cytologically enriched at the Xi in an NF‐κB‐dependent fashion [123]. Despite the absence of Xist RNA and repressive histone modifications in naïve lymphocytes, the Xi is mostly transcriptionally silent [124]. The epigenetic mechanisms that regulate the dynamic recruitment of epigenetic modifications while preserving XCI across the Xi are under active investigation, and recent discoveries have shed light on the key roles of Xist RNA‐independent and dependent deposition of heterochromatic modifications and DNAm marks across the Xi in female lymphocytes, which are summarized below.

**FIGURE 1:**
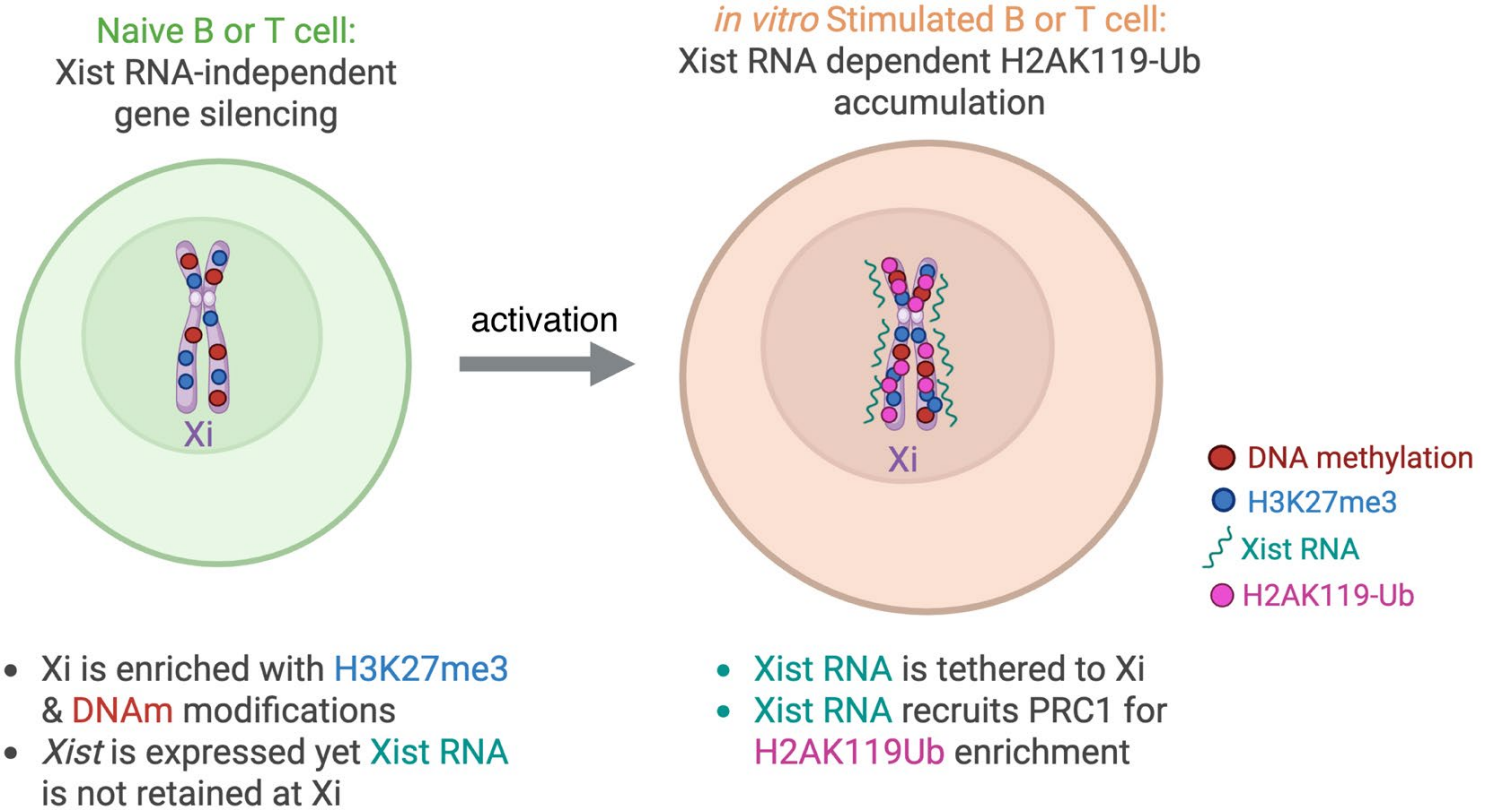
Epigenomic changes across the Xi in naïve and in vitro stimulated lymphocytes. In naïve T and B cells, the Xi is mostly transcriptionally repressed yet expresses *Xist* and contains H3K27me3 (blue circles) and DNA methylation (DNAm; red circles) marks, likely functioning as imprints of transcriptional repression from XCI initiation silencing in the female embryo. Following in vitro stimulation, Xist RNA (green wavy lines) recruits PRC1 for deposition of H2AK119‐Ubiquitin (Ub; pink circles) marks and additional H3K27me3 marks across the Xi.

Most genes on the Xi in lymphocytes are repressed, yet *Xist* and about 40–50 X‐linked genes are expressed from this chromosome, and some Xi‐linked genes exhibit cell state‐specific expression patterns [124]. Recent work using allele‐specific epigenomic profiling has revealed that the Xi in naïve B and unstimulated T cells is epigenetically marked with H3K27me3 and DNA methylation (Figure 1) [55, 123]. Surprisingly, H3K9me3 levels are higher on the Xa than the Xi in naïve B cells, and stimulation does not alter H3K9me3 accumulation across the Xi, which is unlike female fibroblasts, which exhibit high levels of H3K9me3 on the Xi, in particular gene‐poor regions [62]. In vitro stimulation of female T and B cells results in significant increases of H3K27me3 and H2AK119‐Ub levels at promoters, gene bodies and intergenic regions across the Xi (Figure 1) [55, 123]. Unlike the mechanism for Xist RNA‐mediated XCI initiation, where PRC1 deposits H2AK119‐Ub modifications before H3K27me3 deposition by PRC2 [95], B cell stimulation triggers H2AK119‐Ub marks to be added at specific sites pre‐marked by H3K27me3 imprints [55]. The recruitment of PRC1 to the Xi requires Xist RNA in B cells, yet H3K27me3 enrichment is mostly Xist RNA independent, unlike XCI initiation and maintenance in fibroblasts [95].

Using allele‐specific X‐linked probes for fluorescence imaging and high‐throughput chromatin conformation capture (Hi‐C), the global territory and compartmentalization of the Xi and Xa in B cells are similar and less compact compared to female fibroblasts [124]. While the Xi compaction does not change with B cell stimulation, there are dynamic changes with topologically associated domains (TADs) across the Xi [124]. *Xist* deletion during B cell development using Mb1‐Cre Recombinase mice (Xist^1lox/mb1+^) changes TAD boundaries and X chromosome compaction in mature CD23+ B cells, yet X‐linked gene expression does not change in naïve and in vitro stimulated cells [124]. The TAD boundary changes resulting from *Xist* deletion are likely due to the loss of H3K27me3 and H2AK119Ub modifications across the Xi, observed in B cells from Xist^1lox/mb1+^ female animals [55]. Surprisingly, when *Xist* is deleted ex vivo immediately prior to stimulation, H3K27me3 marks can still accumulate on the Xi, because pre‐existing low levels of H3K27me3 enrichment on the Xi in naïve B cells can recruit PRC2, leading to the continuous accumulation of this mark. However, H2AK119 Ub accumulation does not occur across the Xi for Xist in vitro deleted B cells, indicating that Xist RNA guides PRC1 for H2AK119‐Ub accumulation upon B cell stimulation (Figure 1).

Naive lymphocytes are quiescent, and cellular activation releases NF‐κB protein to enter the nucleus for transcriptional activation genome‐wide [125]. T cell Receptor (TCR) engagement, specifically NF‐κB activation, is necessary for Xist RNA recruitment and H2AK119Ub enrichment at the Xi [123]. Either chemical inhibition or genetic deletion of signaling pathways upstream of NF‐κB activation prevents Xist/XIST RNA accumulation at the Xi and altered X‐linked gene expression at both the Xi and Xa chromosomes [123]. This novel connection between NF‐κB signaling and XCI maintenance may be a factor in female‐biased autoimmune diseases such as systemic lupus erythematosus (SLE) and some rheumatic diseases, as autoimmune patient lymphocytes often exhibit aberrant NF‐κB activation and chronic inflammation [126, 127].

## 5 Female‐Biased Systemic Autoimmune Diseases

Systemic autoimmune diseases are characterized by immune dysfunction and loss of self‐tolerance mechanisms, resulting in chronic, aberrant inflammation and systemic tissue damage [128]. Patient lungs and kidneys are often affected, which correlates with poorer disease prognosis and patient death [128]. The prevalence rates of autoimmune conditions are growing with yearly increases in global incidence and prevalence of 19.1% and 12.5%, respectively [129]. The exact etiology of these diseases remains elusive, but growing research suggests that a combination of genetic, endocrine, infectious, and environmental factors contributes to the loss of self‐tolerance [130]. Females are overwhelmingly more affected by autoimmune diseases compared to males. While sex hormones play an important role in the female bias, there is also evidence that multiple X chromosomes increase the risk of developing some female‐biased autoimmune diseases [131]. Multiple X chromosomes is associated with higher risk for Systemic Lupus Erythematosus (SLE) [132, 133], Systemic Sclerosis (SSc) [134], and Sjogren's syndrome [135], and this increased risk is sustained even in male patients with Klinefelter's syndrome (XXY), suggesting a clear role for increased X‐linked immune gene dosage in these conditions [131].

SLE is a heterogeneous autoimmune disease whose clinical presentation varies drastically depending on the patient's age, ethnicity, and sex [136]. Regardless of clinical manifestation, all SLE patients harbor multisystem microvascular inflammation and high levels of antinuclear autoantibodies [136]. SLE is one of the most female‐biased systemic autoimmune diseases, where 85%–90% of SLE patients are female [137]. North America has the highest incidence and prevalence of SLE, whereas Africa and Australia have the lowest reported incidence and prevalence, respectively [138]. Within the United States, higher frequencies of lupus are observed in African American, Hispanic, and Asian women [138] of childbearing age (15–44 years) [139]. African American women are 3–4 times more likely to be diagnosed with the disease than Caucasian women of the same age [139]. Rheumatoid Arthritis (RA) is an autoimmune disease that causes chronic inflammation in the synovium, erosion of bone and cartilage, and progressive degradation of the joints [140]. Females are 2–3 more likely to develop RA [141] and tend to experience more aggressive disease progression and poorer prognoses compared to male RA patients [142]. SSc is another systemic autoimmune disease that affects the connective tissues [143], exhibiting variability with disease severity and affected organs [143], and SSc has one of the highest mortality rates of all rheumatic diseases [144, 145]. The hallmark feature of SSc is progressive fibrosis resulting from aberrant deposition of extracellular matrix components in tissues and organs throughout the body [146], vascular pathologies and autoimmune phenotypes [143]. Incidence and prevalence rates of SSc are difficult to accurately measure based on the rarity and heterogeneity of the disease, yet recent reports indicate incidence rates of 16.4 people per 100,000 people/year and a prevalence of 24.4 people per 100,000 people/year [147]. SSc also exhibits a female bias, and this bias is more pronounced for systemic versus limited SSc [148, 149, 150, 151, 152, 153]. There are gene and environment interactions that contribute to SLE, RA, and SSc pathogenesis [139, 140, 141], which include UVB radiation, pathogen infection [154], heavy metals, smoking, drug use/exposure [143], and asbestos exposure, some of which are associated with increased disease activity [155]. While the mechanisms underlying environmental exposures and autoimmune disease onset/activity are not well understood, exposure appears to increase levels of autoreactive adaptive lymphocytes, production of self‐antigens, autoantibodies, cell death and release of immune complexes that infiltrate target organs [138, 156].

IFN‐α, a Type I interferon (IFN), has been implicated as a driving factor for several female‐biased autoimmune diseases including SLE and SSc [157]. About 50% of SLE patients have elevated serum IFN‐α that directly correlates with disease activity and severity [158]. Recent studies suggest estrogen can modulate Type I IFN activity [159], as SLE symptoms are aggravated by estrogen in certain mouse models of SLE [160]. Female SLE patients have significantly higher levels of estrogen metabolites and lower progesterone metabolites than male SLE patients [161, 162]. In vitro studies of dendritic cells (DCs) exposed to estrogen have significantly increased production of pro‐inflammatory cytokines [163], and progesterone treatment in mice can block TLR7‐mediated IFN‐α production by pDCs [164]. Type I IFN production is also influenced by the X chromosome, as TLR7 and TLR8 are both encoded on the X chromosome [159], and exhibit variable XCI escape in pDCs of SSc patients [165], which can increase type I IFN and elevate ISG expression, which is discussed in the next section. Thus there are both hormonal and X‐linked contributions for the striking female bias with some autoimmune diseases, and more work is necessary to investigate how these factors influence the female bias across single‐organ and systemic autoimmune diseases.

## 6 Dosage‐Sensitive X‐Linked Immune‐Related Genes in Autoimmune Diseases

The X chromosome contains a high density of immune‐related genes [47], and recent work indicates that many of these X‐linked genes are aberrantly expressed in immune cells from patients with autoimmune diseases such as SLE and SSc [122, 166, 167]. There are also GWAS studies investigating the genetic susceptibility for autoimmune disease, which have reported abnormalities in the dosage and function of some X‐linked immune genes [168]. In this section, we highlight some dosage‐sensitive, immune‐related X‐linked genes associated with autoimmune diseases that exhibit a female bias.

### 6.1 Toll‐Like Receptor 7 (TLR7)

Toll‐like receptor 7 (TLR7) is a Pattern Recognition Receptor (PRR), which is essential for the innate immune system to detect pathogenic microorganisms through its ability to bind to single and double‐stranded DNA and RNA [169]. There are many classes of PRRs, including TLRs that are crucial moderators of the innate inflammatory immune responses [170, 171]. In humans, about 10 functional TLRs have been reported, whereas up to 12 TLRs have been described in mice [172, 173, 174]. TLRs are distributed through the cell membrane and endosomal compartments and can bind to single or double‐stranded nucleic acids [169]. TLRs activate downstream signaling cascades, which can amplify the original signal and also increase transcription of pro‐inflammatory and antiviral factors [175, 176, 177]. *TLR7* is an X‐linked gene that exhibits variable XCI escape in B cells, pDCs, and monocytes [167, 178], and TLR7 protein is localized to the endosomal membranes [169]. The ligand‐binding domain of TLR7 recognizes single‐stranded RNA fragments [179] which increase production of pro‐inflammatory cytokines including TNF‐a, IL‐1, and IL‐6, along with type I and type II interferons (IFNs) [179] through activation of the MyD88 pathway. Under normal biological circumstances, activation of TLR7 by self‐antigens is prevented through receptor localization to endosomal membranes [180, 181]. However, nucleic acids can serve as dual specificity antigens through their ability to activate nucleic acid sensitive B cell receptors (BCRs) and cause receptor‐mediated endocytosis to TLR7‐containing endosomal compartments [182].

*TLR7* is an example of a dosage‐sensitive X‐linked gene where overexpression or increased protein activity is associated with autoimmune disease. Increased B cell TLR7 signaling increases nuclear autoantibody production and the eventual loss of tolerance [182]. Increased TLR7 signaling has been reported in SLE patients and various mouse models of lupus‐like disease [182, 183, 184, 185, 186, 187, 188]. Altered dosage of *Tlr7* results in lupus‐like phenotypes in mice, as the *Yaa* mouse model [189] contains a Y‐linked autoimmune accelerating (Yaa) locus consisting of 16 X‐linked genes, including *Tlr7* [190], translocated onto the Y chromosome [189, 190]. Lupus‐like phenotypes can be ameliorated by deletion of *Tlr7* in various lupus mouse models [182]. Recent clinical studies indicate that SLE patients can have increased TLR7 mRNA that is positively correlated with high IFN‐a mRNA levels in PBMCs [191]. Monogenic mutations in *TLR7* in SLE patients have been reported [184, 185, 186], and a TLR7 mutation (TLR7Y264H) was recently identified in SLE patients that increases both the affinity to endogenous ligands and amplifies TLR7 signaling pathways, resulting in B cell‐mediated production of self‐antigens [192]. Additional work is necessary to determine the fate of biallelic *Tlr7* B cells in autoimmune disease to reveal whether increased *Tlr7* expression can impact loss of tolerance and if biallelic *Tlr7* expressing cells undergo cell death to be removed from circulation.

### 6.2 CD40 Ligand (CD40LG)

CD40LG is a transmembrane glycoprotein that is primarily expressed on platelets and activated T cells, but can also be found on other immune cells such as NK cells, monocytes, and B cells during inflammatory conditions [193, 194]. CD40LG on activated T cells engages with CD40 receptors on B cells acting as a costimulatory stimulus to activate an adaptive immune response [195]. In the absence of this costimulatory stimulus, activation and priming can still occur in B and T cells, respectively, but many cell and immune functions are severely impaired [196]. Overexpression of *CD40LG* is a feature of SLE patient T cells, and often *CD40LG* expression levels directly correlate with disease severity [197]. T cell overexpression of *CD40LG* results in aberrant B cell activation, which has been demonstrated using B cell tumor models [197]. *CD40LG* can be aberrantly expressed in both monocytes and B cells of SLE patients, resulting in CD40L‐dependent spontaneous antibody production [198]. Transgenic mice with increased *Cd40lg* expression exhibit chronic inflammation, glomerulonephritis and spontaneous anti‐DNA autoantibody production [199]. Patients with a *CD40LG* gene duplication also have features of autoimmune disease [200]. The *CD40LG* gene exhibits variable escape from XCI in human fibroblasts [201], and *CD40LG* expression is influenced by DNA methylation enrichment within the promoter region. Treatment of female CD4+ T cells with DNA methylation inhibitors results in increased expression of *CD40LG* [202], suggesting that *CD40LG* could become reactivated from the Xi in human cells. However direct evidence for *CD40LG* reactivation specifically from the Xi, and whether reactivation of mouse *CD40lg* from the Xi occurs in T cells, is unknown.

### 6.3 Bruton's Tyrosine Kinase (BTK)

Bruton's tyrosine kinase (BTK) regulates the B cell receptor (BCR) signaling pathway and is important for the regulation of tolerance pathways [203, 204, 205, 206] and humoral immunity [207]. BTK activates BCR signaling cascades, yet BTK can also regulate CD40, FcR, and chemokine receptor pathways and also TLR signaling networks in B cells and myeloid cells [208, 209, 210, 211, 212, 213, 214, 215, 216]. In mouse models, *Btk* overexpression results in SLE phenotypes, with the development of spontaneous germinal centers and autoantibody formation [205]. Gain‐of‐function Btk mutations that result in constitutive Btk signaling activity can generate autoreactive IgM plasma cells in mice [217]. However, loss‐of‐function Btk mutations can ameliorate autoantibody production and autoimmune phenotypes in *Lyn*‐deficient mice [203]. *BTK* is normally silenced on the Xi in human fibroblasts [201], yet it is overexpressed in PMBCs of patients with lupus nephritis [218], suggesting that some immune cells may have reactivated *BTK* from the Xi. Additional work is necessary to determine whether *BTK* can become reactivated during chronic inflammation or autoimmune disease.

### 6.4 Xist RNA

**FIGURE 2:**
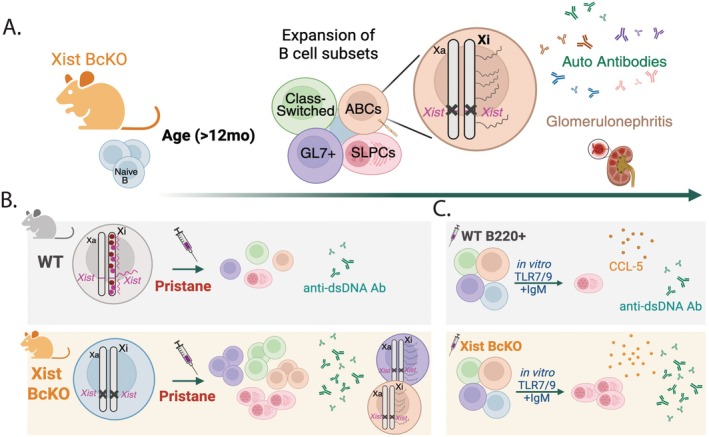
Autoimmune phenotypes resulting from a B cell deletion of Xist RNA. (A) Female mice with a B‐cell‐specific deletion of Xist RNA (Xist BcKO) on a BALB/c background can develop autoimmune phenotypes with advanced age. Some aged female Xist BcKO mice with elevated anti‐dsDNA autoantibodies (Ab) also exhibit: expansion of activated GL7+ B cells, class‐switched B cells, age‐associated B cells (ABCs), and short‐lived plasma cells (SLPCs); altered X‐linked gene expression in ABCs; diversity of autoantibodies associated with female‐biased autoimmune diseases; glomerular pathologies. (B) Pristane injection of Xist BcKO female mice results in greater expansion of B cell subsets, increased anti‐dsDNA autoantibody production, and altered X‐linked gene expression in ABCs and GL7+ activated B cells compared to wildtype (WT) female mice. (C) In vitro culture of B220+ B cells from pristane‐treated Xist BcKO and WT female mice with TLR7/9 agonists generates more plasma cells, anti‐dsDNA Ab, and increased levels of the cytokine CCL5.

Xist RNA, expressed exclusively from the Xi, is essential for forming the Xi during development and also important for maintaining XCI along with various heterochromatic epigenetic modifications (reviewed in the previous section). Recent evidence suggests that Xist RNA and XCI maintenance can influence immune cell function. Cell‐type specific *Xist* deletions result in variable phenotypes depending on the developmental stage and tissue of the deletion, where ~3–50 genes on the Xi can become reactivated, resulting in subtle yet significant X‐linked dosage changes of ~1.015–1.36 [131]. Deletion of the long noncoding RNA Ftx, which is an activator of *Xist* expression, reduces Xist RNA levels by ~50% in immune cells and aged Ftx KO female mice develop autoimmune phenotypes [219]. Aged female Ftx KO mice develop elevated levels of anti‐dsDNA and anti‐Smith RNP autoantibodies, and expansion of monocytes, pDCs, activated T and B cells. X‐linked gene expression is not altered in immune cells of Ftx KO mice lacking autoimmune phenotypes, yet aged Ftx KO mice have immune‐cell specific upregulation of ~2–10 X‐linked genes including *Tlr7, Tlr13*, *Cxcr3*, *Cfp*, and *Tasl* [219]. *Xist* deletion in B cells using Mb1‐Cre Recombinase mice (Xist BcKO) also results in the spontaneous development of autoimmune phenotypes, including SLE‐associated autoantibodies and expansion of activated B cells, atypical B cells, and plasma cells (Figure 2) [220]. Female Xist BcKO mice treated with pristane produce significantly higher amounts of anti‐dsDNA autoantibodies and expanded activated B cell subsets compared to wildtype treated female mice (Figure 2) [220]. Comparison of X‐linked differentially expressed genes in atypical B cells from pristane‐treated and spontaneous diseased Xist BcKO mice reveals ~8–10 X‐linked genes, including *Tasl, Cfp*, and *Ftx* [220]. *Xist* deletion prevents H3K27me3 and H2AK119‐Ub accumulation on the Xi in activated B cells [55], and there are fewer H3K27me3 marks across the X chromosome in B cell subsets of pristane‐treated mice, including at the *Tasl* promoter [220]. *Xist* deletion favors B cell activation and differentiation into plasma cells, and in vitro culture experiments demonstrate that B cells from pristane‐treated Xist BcKO mice produce more anti‐dsDNA autoantibodies and the chemokine CCL5 (Figure 2). Thus, reduction or deletion of Xist RNA can alter XCI maintenance and the expression of some X‐linked immunity‐related genes, resulting in expansion of activated immune cells and autoimmune phenotypes.

Recently, it has been proposed that Xist RNA itself can contribute to the female bias of autoimmune disease. Xist RNA contains UU dinucleotides and a TLR7‐specific binding motif, suggesting that regions of the Xist RNA could function as TLR7 ligands for activating immune cell signaling pathways (reviewed in the next section). Short XIST RNA sequences incubated with pDCs activated TLR7 signaling and type I IFN production [221]. The study suggests that the release of XIST RNA from dying cells becomes an available ligand for pDCs and B cells, thereby increasing the type I IFN signaling pathway. Additional work is needed to test this hypothesis in vivo. Xist RNA‐protein complexes have also been proposed as a source of autoantigens. Using a doxycycline‐inducible Xist cDNA transgene lacking the Repeat A region (Xist TgA‐), male Xist TgA‐ mice were treated with pristane and developed some features of lupus‐like disease with similar severity as pristane‐treated wildtype female mice [222]. Autoantibodies against Xist RNA‐associated binding proteins were detected in serum of male pristane‐treated Xist TgA‐, including serum of male and female SLE, SSc, and dermatomyositis patients [222]. In sum, these two studies identify novel pathways where Xist RNA directly impacts type I IFN signaling and may act as a “scaffold” for autoantigen production in the context of autoimmune disease. Additional work is needed to investigate the role for Xist RNA for loss of tolerance and how it functions as a template for specific Xist RNA‐protein complexes that eventually become autoantibodies in vivo.

## 7 Sex Differences in Cell Signaling Pathways

Sex differences in immune cell signaling pathways also influence disease susceptibility and responses to immune therapies. Signaling pathways are essential for coordinating host responses to pathogens and mitigating tissue injury, and their regulation shapes both protective and pathological outcomes. Evidence for sex differences with signaling pathways has been observed across a range of conditions, including autoimmune diseases such as systemic lupus erythematosus (SLE), viral infections such as HIV and influenza, and cardiovascular pathologies where men often demonstrate greater morbidity. These differences with signaling pathways help explain differences in disease prevalence and severity as well as variability in the efficacy of vaccines and responses to immunotherapies. Understanding mechanistic differences with male and female signaling pathways can contextualize sex‐specific disease prevalence and guide future sex‐specific therapies. In this section, we review the sexually dimorphic regulation of type 1 interferon signaling, particularly via the X‐linked TLR7 pathway, as well as cytokine and growth factors, JAK/STAT, and NF‐κB pathways.

### 7.1 Female Bias With Type I Interferon Production and the X‐Linked TLR‐7 Protein

Type I interferons (IFN‐I) are a family of cytokines that are produced by immune cells in response to viral and pathogen infections. The Type I IFN family consists of IFN‐α, IFN‐β, IFN‐ε, IFN‐κ, and IFN‐ω. Of these, IFN‐α and IFN‐β are the most studied, with 13 different genes for IFN‐α that share 70%–99% amino acid sequence identity, yet there is just 1 IFN‐β gene. There is less information about IFN‐ε, IFN‐κ, and IFN‐ω, although emerging evidence suggests these may have tissue‐specific or sex‐dependent roles. Type I IFNs are produced when pattern recognition receptors bind pathogen or host nucleic acids, and IFN‐I molecules can bind to type I IFN receptor (IFNAR), which is on the surface of many immune cells [223]. IFNAR engagement results in immune cell activation, which can reduce viral replication and spreading, and also enhances antigen presentation, promotes T and B cell activation, and cellular differentiation [224]. Type I IFNs are required for host defense pathways and immune surveillance, and their activity must be carefully regulated as constant or excessive signaling results in chronic inflammation, a feature of autoimmune disease.

X‐linked TLR‐7 (reviewed in a previous section) is a pattern recognition receptor on endosomes that recognizes single‐stranded RNAs and induces type I IFN production [225, 226]. There are sex differences with TLR7‐driven interferon responses, influenced by both sex hormones and genetics. Female pDCs stimulated with TLR7 agonists result in significantly greater IFN‐α production compared with male cells [227]. Female macrophages from women infected with HIV produce higher levels of interferon signature gene (ISG) transcripts when normalized to viral load [228, 229]. Female cells also exhibit greater NK cell activation downstream of type I IFN [230]. TLR7 can escape XCI in some healthy female B cells, plasmacytoid dendritic cells (pDCs), and monocytes, resulting in higher protein levels and altered immune cell function [225]. Estrogen can also increase TLR7 signaling and type I IFN production by pDCs in vivo [231]. Androgens can suppress type I IFN [230]. Taken together, the data indicate that females mount a more robust type I IFN response at both the transcriptional and functional level, providing enhanced antiviral protection but also increasing susceptibility to interferon‐driven autoimmune diseases.

### 7.2 Sex Differences With Cytokine and Growth Factor Pathways

Cytokines and growth factors coordinate the proliferation, survival, and differentiation of immune cells, while also integrating signals from the tissue microenvironment. Cytokines are small molecules that are either pro‐inflammatory or anti‐inflammatory and are responsible for mediating immune cell communication in response to inflammation and immune responses from innate and adaptive immune cells. Examples of pro‐inflammatory cytokines include interleukin‐1 (IL‐1), tumor necrosis factor (TNF), interferon‐gamma (IFNγ), IL‐12, IL‐18, and the granulocyte‐macrophage colony‐stimulating factor (GM‐CSF). The anti‐inflammatory cytokines include IL‐4, IL‐10, IL‐13, IFNα, and the transforming growth factor (TGF)β. Ultimately, cytokine signaling activates downstream nuclear transcription factors such as NF‐κB, Smad, and STAT proteins that activate various gene regulatory networks [232].

Cytokines and growth factor proteins serve as central communication centers, shaping both protective immune responses and pathological inflammation. Granulocyte‐Macrophage Colony‐Stimulating Factor (GM‐CSF) is a cytokine that acts as a growth factor to stimulate the production of monocytes, macrophages, and granulocytes, which exhibit sex differences with cell number. Other examples of growth factors include vascular endothelial growth factor (VEGF), epidermal growth factor (EGF), and platelet‐derived growth factor (PDGF), as well as steroid sex hormones estrogen, androgen, and progesterone [233]. Males and females activate growth factor and cytokine pathways differently, resulting in differences with downstream receptor‐mediated signaling that results in varied immune phenotypes. Examining these networks therefore provides a crucial lens for understanding how sex shapes immune function beyond interferon biology.

Sex differences in cytokine and growth factor signaling have been reported in cardiovascular pathology from patients with coronary artery disease (CAD). Monocytes from female CAD patients have higher levels of EGF, IFN‐1, GM‐CSF, VEGFA, and expression of X‐linked CD40LG protein, whereas male patients show higher levels of insulin, HMGB1, and IL‐4 [234]. Serum cytokine profiling indicates that some male CAD patients have significantly lower levels of IL‐2 and IFN‐γ and higher levels of MCP‐1 compared to females, whereas other cytokines showed no sex‐specific differences [235]. While female CAD patients generally exhibit less atherosclerosis, their IFN‐γ levels are typically elevated, reflecting more inflammation despite less cardiovascular pathology. These findings suggest that males and females may utilize distinct immune and growth factor networks in response to vascular stress, which could contribute to sex‐specific patterns of disease progression and outcomes. Female‐specific increases with type I IFN and growth factor–related pathways may reflect a greater reliance on antiviral and tissue‐repair signaling, whereas males may rely more on metabolic and stress‐response pathways such as insulin and HMGB1 signaling. While these observations provide valuable insights for sex differences with cytokine and growth factor pathways, more investigation is necessary to determine how these pathways are influenced by sex in other disease contexts such as autoimmune disease or auto‐inflammatory conditions.

### 7.3 Sex Differences With JAK/STAT Signaling Pathways

Cytokine and growth factor pathways ultimately converge on shared intracellular cascades, and most pathways lead to the activation of Janus Kinase (JAK), which activates the JAK/STAT pathway that regulates cell proliferation, differentiation, cell survival, and immune cell activation [236]. JAK activation results in phosphorylation of STAT proteins, which dimerize then translocate into the nucleus and regulate gene expression. Thus, extracellular cues can quickly induce nuclear transcriptional programs for gene regulation, impacting both normal cellular functions and disease outcomes [237]. The JAK family consists of JAK1, JAK2, JAK3, and TYK2, which phosphorylate tyrosine residues of STAT proteins, cytokine receptors such as interleukins and interferons, and can also phosphorylate JAK proteins themselves for auto‐activation [238]. The STAT family of proteins includes STAT1, STAT2, STAT3, STAT4, STAT5a, STAT5b, and STAT6, where each protein exhibits distinct regulatory functions for finely‐tuned responses to an array of cytokines and growth factors [239]. Aberrant STAT3 activation is associated with malignancy and autoimmunity [240], whereas JAK2 hyperactivation is implicated in myeloproliferative disorders [241]. The JAK/STAT pathway plays a pivotal role in the pathogenesis of numerous disease conditions, including autoimmune diseases, inflammatory disorders, and cancer, where dysregulation of JAKs or STATs can amplify activation pathways [242]. Because various cytokine and growth factor networks converge on JAK/STAT, it has been a focus for therapeutic interventions for autoimmune and inflammatory diseases, many of which exhibit sex differences [243]. There is emerging evidence suggesting that JAK/STAT signaling is modulated in a sex‐specific manner. In ulcerative colitis, male patients exhibited higher phosphorylation of JAK2 and STAT3 compared to female patients, yet JAK1 phosphorylation was similar across both sexes, reflecting sex‐specific selective regulation of pathway components [244]. In the autoimmune diseases RA and SLE, STAT4 and STAT5 have been implicated in sex‐biased disease pathogenesis, as these STATs are expressed at higher levels in females [245, 246]. However, additional work is necessary to examine whether STAT4/5 exhibit sex‐specific regulation in autoimmune disease contexts.

### 7.4 Sex Differences With the NF‐κB Pathway

The nuclear factor kappa‐light‐chain‐enhancer of activated B cells (NF‐κB) pathway is another central signaling cascade through which cytokines, growth factors, and pathogen‐associated stimuli orchestrate immune responses. NF‐κB is a master activating switch for inflammatory gene expression capable of starting both innate and adaptive immune responses but also cell proliferation, survival, and apoptosis [247]. Inactive NF‐κB dimers are sequestered in the cytoplasm bound to IκB proteins, creating a poised but restrained system. Engagement of T cell or B cell receptors, toll‐like receptors, cytokine, and antigen receptors rapidly triggers IκB phosphorylation then degradation, freeing NF‐κB subunits which translocate into the nucleus where they activate a broad repertoire of target genes [248]. This “ready‐to‐fire” configuration allows immune cells to convert external cues into activation of large transcriptional programs within minutes, shaping both protective and pathological outcomes. Perturbations NF‐κB regulation have been implicated in a wide spectrum of conditions, including autoimmunity, chronic inflammatory disorders, viral infections, cancers, and aberrant apoptosis, where constitutive activation of NF‐κB amplifies pathological inflammation, promotes malignant transformation, and alters cell death pathways [249]. The NF‐κB signaling pathway is a centralized downstream hub where diverse stimuli converge, making it a critical target for understanding and modulating disease processes.

Recent work suggests that NF‐κB activation pathways may be modulated in a sex‐specific manner. Estrogen can disrupt NF‐κB mediated transcriptional activation by inhibiting the formation of a complex between MyD88 and a methylated form of ERα complex that signals for inflammation [250]. In glioblastoma cell culture models, female‐derived cells exhibit enhanced senescence and reduced NF‐κB activation compared to male‐derived cells [251], and it is intriguing to consider that sex differences in NF‐κB activity in glioblastoma alter cellular function and influence disease outcomes. Additional work using mouse models is needed to determine if the sex differences with NF‐κB activation also occur in reproductive cancers. Given the novel connection between NF‐κB activation and regulation of XCI maintenance in female T cells (discussed in a previous section), it is exciting to consider X‐linked pathways that regulate NF‐κB activation, specifically through XCI escape genes such as *XIAP* or *IKBKB* (*NEMO*) in the context of sex differences. While most of the literature describing sex differences with NF‐κB signaling is from oncologic and neurologic contexts, additional work investigating whether NF‐κB activation exhibits sexual dimorphism in autoimmune or systemic inflammatory diseases is unknown.

## 8 Sex‐Based Viral and Vaccine Responses in Influenza, SARS‐CoV‐2, and HIV

Biological sex also influences immune responses to viral pathogens at multiple levels, ranging from tissue‐intrinsic programming to systemic host‐pathogen interactions. Because of sex differences with innate and adaptive immune responses, this also impacts viral clearance, viral‐induced disease severity, and vaccine responsiveness [4, 17]. Sex differences following viral infection are influenced by multiple factors including sex hormone signaling, strength of type I interferon responses, and X‐linked immune gene expression, and recent work on some of these pathways is highlighted in the following sections.

### 8.1 Influenza

Studies of influenza infection have consistently demonstrated sex differences in both humoral and cellular immunity that vary with age. Prepubescent males have more severe disease after flu infection compared to age‐matched females, and males of advanced age and perimenopausal females exhibit more severe illness and lung pathologies [252]. Estrogen plays an important role in mediating these differences by modulating B and T cell development, cellular trafficking, and regulating the production of key cytokines and chemokines, thereby enhancing antiviral responses in females [253]. The administration of 17β‐estradiol (E2) following ovariectomy in female mice restores protective immune responses, suppressing inflammatory markers such as TNF‐α and CCL2 compared to ovariectomized‐only and sham‐intact controls [254]. These findings underscore the dual role of estrogen in promoting effective immunity while limiting excessive inflammation.

Sex differences with responses to influenza infection are also recapitulated in mouse models. Adult female C57BL/6 mice infected with H1N1 or H3N2 viral strains have lower LD50 values compared to males, along with greater losses of body mass and more hypothermia at median infectious doses [255]. Female mice exhibit greater IgG2c class switching in response to influenza vaccination compared to males, resulting in enhanced antibody production [256]. Similarly, injection of inactivated influenza virus in female mice results in increased numbers of germinal center B cells and elevated CD4+ and CD8+ T cell populations [257], reflecting more potent memory and recall responses. Female mouse splenocytes show enhanced antigen presentation and lymphocyte activation in response to influenza infections in both in vivo and in vitro settings [258]. Female mice infected with H1N1 exhibit stronger induction of proinflammatory cytokines and chemokines including CCL2, TNF‐α, IFN‐γ, and IL‐6 1 week post‐infection [259].

Female‐specific heightened immune responsiveness is also observed after vaccination to influenza. Females experience a higher incidence of vaccine‐associated adverse effects, including local and systemic reactions [2, 260, 261, 262, 263, 264]. These observations have prompted discussion of sex‐specific vaccination strategies, such as adjusting dose or scheduling to optimize efficacy while minimizing reactogenicity in females. Influenza vaccines thus provide an example of how intrinsic sex differences with immunity confer both enhanced protection and potential adverse effects.

Because infant and pre‐pubertal males exhibit higher rates of morbidity after influenza infections [265], the genetics of the sex chromosomes likely play an important role in sex differences. While the identification of critical X‐linked genes and immune cells responsible for these sex differences is unclear, there is evidence for some X‐linked genes, such as *Tlr7*, that contribute to female‐biased protection following influenza infection. *Tlr7* exhibits variable XCI escape and can result in higher expression in B cells which promotes class‐switch recombination to IgG2c, which enhances humoral responses against influenza [266, 267]. X‐linked mechanisms likely act synergistically with hormonal regulation to establish robust female‐specific immune responses of rapid antibody production, and unraveling these pathways will likely yield exciting connections between hormones and X‐linked genes responsible for sex‐biased outcomes from influenza infections.

### 8.2 SARS‐CoV‐2

The global spread of COVID‐19 provided one of the clearest demonstrations that biological sex significantly influences both the course of viral infection and the profile of vaccine‐induced immunity. Epidemiological data consistently indicate that males are at a higher risk of severe disease and mortality from SARS‐CoV‐2, a pattern attributed in part to poorer CD8+ T cell activation and less robust adaptive immune responses [268]. In contrast, females more frequently mount stronger antiviral responses but also report higher rates of post‐vaccination reactogenicity [269, 270]. Emerging evidence implicates sex hormones and X‐linked factors in differential susceptibility and severity. Estrogen downregulates expression of X‐linked *ACE2*, a key receptor for SARS‐CoV‐2 viral entry, while androgens upregulate expression of *TMPRSS2*, a protease that also facilitates viral entry [270, 271, 272]. These differences with *ACE2* and *TMPRSS2* expression likely contribute to the observed male predominance in severe COVID‐19 [273, 274]. However, there are additional biological mechanisms also involving sex hormones and X‐linked immune genes that influence sex differences with innate immune sensing, divergent T cell and humoral response programs.

The interferon signaling pathways activated by SARS‐CoV2 infection exhibit sex differences. Infected female patients demonstrate higher levels of IFNα2, a type I interferon involved in antiviral defense [269]. In contrast, SARS‐CoV2 infected male patients have higher circulating levels of innate inflammatory cytokines, such as IL‐8, IL‐18, and CCL5, along with expansion of non‐classical monocytes that correlate with worse clinical outcomes and blunted T cell activation, particularly in older adults. These findings suggest that following SARS‐CoV2 infection, males experience a more proinflammatory but less effective antiviral state, whereas females exhibit a more coordinated early innate response which has protective advantages. It is likely that female‐specific increased expression of X‐linked immune genes like *TLR7* and sex hormone regulation of interferon pathways contribute to sex differences following SARS‐CoV2 infection. Estrogen signaling enhances pDC‐mediated IFN‐α production, amplifying downstream antiviral defenses [231, 275, 276]. Macaque models demonstrate that treatment with pegylated IFN‐α protected lung tissue from SARS‐CoV regardless of sex [277], underscoring the critical role for IFN‐α to control viral amplification, and its potential as a therapeutic target in SARS‐CoV‐2 infection, as it likely helps to amplify downstream NK cell and CD8+ T cell activation.

Sex differences are also evident with immune responses to COVID‐19 vaccination. Females typically develop higher IgG titers and more sustained neutralizing antibody responses following both primary and booster vaccination compared to males [278, 279]. Sex differences with responses to COVID‐19 vaccination are influenced by multiple factors including age, prior infection, vaccine platform, and the timing of immunization. Enhanced female immune responsiveness also correlates with higher rates of vaccine‐associated adverse effects. Females exhibit higher rates of thrombocytopenia [225], potentially linked to heightened autoantibody responses to platelet factor 4 and hormonal factors such as oral contraceptive use [280]. Conversely, males show a greater incidence of myocarditis and pericarditis following mRNA vaccination, consistent with broader trends observed across multiple vaccine types [281, 282].

Sex‐based differences also extend into the post‐acute phase of infection. Females are more likely to develop Long COVID, characterized by persistent symptoms such as fatigue, brain fog, myalgia, and dyspnea [283, 284, 285, 286]. One study indicates higher IL‐6 levels and fatigue in females, whereas shortness of breath was more male‐biased but not associated with elevated IL‐6 [284]. Yet another study indicates that females have greater autoantibody responses following asymptomatic infection, while males show greater reactivity after mild symptomatic disease [287]. These findings suggest that stronger early immunity in females does not uniformly translate to reduced long‐term morbidity, highlighting that sex‐specific mechanisms of post‐viral immune dysregulation warrant further study.

### 8.3 Human Immunodeficiency Virus (HIV)

Human Immunodeficiency Virus (HIV) infection can progress to Acquired Immunodeficiency Syndrome (AIDS) and also exhibits sex differences with immune responses and disease progression. Females tend to have significantly lower plasma viral HIV loads during early and chronic infection compared to males [288, 289, 290, 291, 292, 293, 294, 295, 296]. This observation is supported by lymph node biopsies, which demonstrate a lower tissue viral burden in females [297]. Paradoxically, however, some females progress to AIDS at faster rates than males despite having lower viral loads [290]. Sex differences in HIV pathogenesis are driven not only by virological factors but also by immunological and inflammatory mechanisms.

Innate immune programming plays a key role in observed sex differences with viral infection and immune responses to HIV. As with COVID‐19, female pDCs produce substantially higher levels of type I IFN‐α following stimulation with HIV‐1‐derived TLR7 ligands or inactivated virus compared to male cells, reflecting sex differences with TLR7 signaling pathways [298]. Oral contraceptive use is not associated with differences in IFN‐α secretion, but rather with the frequency of IFN‐α+ pDCs following stimulation, suggesting that sex hormones may fine‐tune rather than override genetic dosage effects. Enhanced Type I interferon production may contribute to lower viral levels in females, but also to greater immune activation and inflammation, which are well‐established drivers of HIV disease progression independent of viral load. The diagnostic and prognostic algorithms which rely on viral load and CD4+ T cell counts may require knowledge of biological sex for interpretations, particularly in untreated individuals. Pharmacokinetics and immune responses are influenced by sex, requiring sex‐specific analyses of drug efficacy, toxicity, and dosing. Thus, research experiments investigating viral infections and vaccine outcomes should utilize sex‐stratified approaches as mechanisms that mediate early viral control in women (e.g., TLR7‐IFN axis) may also drive immune activation at later stages.

## 9 Conclusions and Future Perspectives

There is continued interest in understanding the hormonal and genetic contributions for sex differences with immune responses and immune‐related diseases. As more immunological research studies incorporate sex as a biological variable, the outcomes raise intriguing questions about the origins for the observed sex differences, specifically the contributions from sex hormones, X‐linked gene expression, epigenetic regulation of autosomal/X‐linked immune genes, or a combination of many of these factors. Advances in high‐dimensional flow cytometry, spatial transcriptomics, and single‐cell profiling have increased our understanding of immune cell responses to pathogens and autoimmune diseases. Yet most mechanistic studies rely on murine models, which, while informative, may not fully capture the complexity of human immune regulation, how hormonal fluctuations across the lifespan impact immune responses, and the interaction of hormones with X‐linked gene regulation. Sex‐stratified translational studies comparing human tissue responses, including organoid systems, hold great promise in addition to mouse models. To date, most studies focus on binary sex differences yet often lack additional information regarding age, hormonal status, or reproductive status, which can dramatically influence immune responses and vaccine outcomes. Longitudinal studies examining sex differences with repeat infections or duration of vaccine‐induced immunity are sparse, thus limiting our understanding of how sex might influence long‐term memory responses. Finally, clinical trials should systematically incorporate sex‐stratified analyses of immune pathway inhibitors or vaccine safety and efficacy, which may reveal sex‐specific dosing information to prevent adverse side effects. Addressing these shortcomings is critical to inform precision medicine strategies that maximize protection while minimizing adverse outcomes across diverse populations.

## Funding

This work was supported in part by NIH R01 AI134834 and R01 AI168047 (to M.C.A.).

## Conflicts of Interest

The authors declare no conflicts of interest.

## Data Availability

The authors have nothing to report.
